# Efficacy of individualized homeopathic treatment and fluoxetine for moderate to severe depression in peri- and postmenopausal women (HOMDEP-MENOP): study protocol for a randomized, double-dummy, double-blind, placebo-controlled trial

**DOI:** 10.1186/1745-6215-14-105

**Published:** 2013-04-23

**Authors:** Emma del Carmen Macías-Cortés, Leopoldo Aguilar-Faisal, Juan Asbun-Bojalil

**Affiliations:** 1 Escuela Superior de Medicina, Instituto Politécnico Nacional, Ave. Plan de San Luis y Salvador Díaz Mirón, Casco de Santo Tomás, Distrito Federal, CP 11340, Mexico; 2 Hospital Juárez de México, Secretaría de Salud, Ave. Instituto Politécnico Nacional 5160, Col. Magdalena de las Salinas, Distrito Federal, CP 7760, Mexico

**Keywords:** Perimenopause, Postmenopause, Depression, Homeopathy, Fluoxetine, Hamilton Rating Scale for Depression, Beck Depression Inventory

## Abstract

**Background:**

The perimenopausal period refers to the interval when women’s menstrual cycles become irregular and is characterized by an increased risk of depressive symptoms. Use of homeopathy to treat depression is widespread but there is a lack of clinical trials about its efficacy in depression in peri- and postmenopausal women. Previous trials suggest that individualized homeopathic treatments improve depression. In classical homeopathy, an individually selected homeopathic remedy is prescribed after a complete case history of the patient. The aim of this study is to assess the efficacy and safety of the homeopathic individualized treatment versus placebo or fluoxetine in peri- and postmenopausal women with moderate to severe depression.

**Methods/design:**

A randomized, placebo-controlled, double-blind, double-dummy, three-arm trial with a six-week follow-up study was designed. The study will be conducted in a public research hospital in Mexico City (Juárez de México Hospital) in the outpatient service of homeopathy. One hundred eighty nine peri- and postmenopausal women diagnosed with major depression according to the Diagnostic and Statistical Manual of Mental Disorders, 4th edition (moderate to severe intensity) will be included. The primary outcome is change in the mean total score among groups on the 17-item Hamilton Rating Scale for Depression after the fourth and sixth week of treatment. Secondary outcomes are: Beck Depression Inventory change in mean score, Greene’s Scale change in mean score, response and remission rates and safety. Efficacy data will be analyzed in the intention-to-treat population. To determine differences in the primary and secondary outcomes among groups at baseline and weeks four and six, data will be analyzed by analysis of variance for independent measures with the Bonferroni *post-hoc* test.

**Discussion:**

This study is the first trial of classical homeopathy that will evaluate the efficacy of homeopathic individualized treatment using C-potencies versus placebo or fluoxetine in peri- and postmenopausal women with moderate to severe depression. It is an attempt to deal with the obstacles of homeopathic research due to the need for individual prescriptions in one of the most common psychiatric diseases.

**Trial registration:**

ClinicalTrials.gov Identifier: NCT01635218.

## Background

Major depressive disorder (MDD) is the fourth most disabling medical condition worldwide and it is expected to be ranked second by the year 2020 [1,2]. It is typically recurrent and often chronic [3,4]. MDD is associated with high health care costs [5]. Women are approximately twice as likely to develop MDD as men [4,6]. The perimenopausal period refers to the interval when women’s menstrual cycles become irregular, which generally occurs above the age of 40. Transition to menopause has long been considered a period of increased risk for depressive symptoms, as was demonstrated in the Harvard Study of Moods and Cycles, a population-based prospective study that examined the association between lifetime history of major depression and the decline in ovarian function [6]. According to the Stages of Reproductive Aging Workshop (STRAW), transition to menopause is the period that precedes menopause and it is characterized by variations in cycle length (>7 days different from baseline or ≥2 skipped cycles and an interval of amenorrhea ≥60 days) [7]. The postmenopausal stage is the period that continues after 12 months or more of amenorrhea. Depressive symptoms increase during the transition to menopause [8]. A significant inverse association of follicle stimulating hormone (FSH) with depressive symptoms provides strong corroborating evidence that the changing hormonal milieu contributes to dysphoric mood in this transition period [9]. With menopause, seric levels of FSH usually exceed 40 mU/ml although, given the variability of individual hormone levels, the determination of menopausal status is generally made by clinical history rather than laboratory parameters. Furthermore, other hormones also change their serum concentrations. The decline in estrogen levels that is associated with menopause results in a wide range of symptoms, which include vasomotor symptoms (hot flushes and night sweats) [10].

The Hamilton Rating Scale of Depression (HRSD) and the Beck Depression Inventory (BDI) are two well-known standardized scales to assess depression severity used in trials worldwide. The Greene Climacteric Scale (GS) is also a standardized scale used in the Mexican population. It is intended specifically to be a brief and standard measure of core climacteric symptoms or complaints to be used for comparative and replicative purposes across different types of studies whether they are medical, psychological, sociological or epidemiological in nature. Three separate sub-scales measure vasomotor symptoms, somatic symptoms, psychological symptoms, and an additional probe is related to sexual function. Psychological symptoms can be further sub-divided to measure anxiety and depression [11,12].

Meta-analyses of antidepressant medications have reported only modest benefits over placebo treatment and, when unpublished trial data are included, the benefit falls below accepted criteria for clinical significance [13,14]. Specifically, a meta-analysis of clinical trial data submitted to the US Food and Drug Administration (FDA) revealed a mean drug-placebo difference in improvement scores of 1.8 points on the HRSD [15], whereas the National Institute for Clinical Excellence (NICE) used a drug-placebo difference of three points as a criterion for clinical significance when establishing guidelines for the treatment of depression in the UK. Antidepressants may be effective for severely depressed patients, but not for moderately depressed ones. Moreover, only about 50% of patients with MDD show a response (>50% reduction in baseline symptoms) and only about one in three attain remission (virtual absence of symptoms) within the first eight weeks of treatment [4,16-18].

Homeopathy is one of the most frequently used and controversial systems of medicine. It is based on the ‘principle of similars’; highly diluted preparations of substances that cause symptoms in healthy individuals are used to stimulate healing in patients who have similar symptoms when ill [19]. When a single homeopathic remedy is selected based on a patient’s symptoms picture, it is called ‘classical’ homeopathy [20]. In classical homeopathy, the treatment consists of two main elements: the case history and the prescription of an individually selected homeopathic remedy. The purpose of the homeopathic case history is to ascertain the totality of signs and symptoms of each patient, enabling the selection of an individualized homeopathic medicine [21].

Homeopathic medicines are produced through sequential agitated dilutions in Decimal (D), Centesimal (C) or Quinquagintamillesimal (Q or LM) potencies [21]. C-potencies are prepared by diluting a drop of a parent substance in 99 drops of ethanol followed by agitation of the solution (1 C). This procedure is repeated in consecutive agitated dilutions (2 C, 3 C, 4 C, and so on). Generally, high potencies are prescribed for mental symptoms.

Use of homeopathy to treat psychiatric and menopausal problems is widespread, and the need for more high-quality controlled trials has been identified [22,23]. Meta-analyses and systematic reviews have drawn mixed conclusions as to whether homeopathy is more effective than placebo in general medicine [20,24-28]. The database on studies of homeopathy and placebo in psychiatry is very limited, but results do not preclude the possibility of some benefit. Homeopathy efficacy was found for fibromyalgia and chronic fatigue syndrome, for example [22].

Bordet *et al.* conducted a multi-national prospective non-comparative observational study of homeopathic treatments for hot flushes and suggest that further investigation is justified [29]. Jacobs *et al.* conducted a randomized, double-blind study versus placebo performed over one year with 83 women suffering from breast cancer; patients received either individualized homeopathic treatment or a homeopathic complex or a placebo. This study did not show any significant difference between the three patient groups relative to the severity and frequency of hot flushes although there was a trend in the ‘individualized homeopathic treatment’ group during the first three months of the study [30]. Although depression is one of the most common symptoms in peri- and postmenopause, there is no controlled study of the homeopathic use of individualized treatment in depressive disorders in women at this stage.

Recently, results from a randomized, controlled, double-blind trial indicated that individualized homeopathic Q-potencies were non-inferior to the antidepressant fluoxetine in a sample of patients with moderate to severe depression [21,31]. Responder rates (defined as a decrease of at least 50% from baseline on the Montgomery and Asberg Depression Rating Scale) were higher (homeopathy 84.6%, fluoxetine 82.8%) than those usually found for antidepressants in clinical trials (43% to 75%) [21,31]. However, the efficacy of the individualized homeopathic C-potencies for peri- and postmenopausal women with depression has not been investigated.

### Aims

The primary aim of the study is to assess the efficacy of individualized homeopathic treatment (IHT) versus fluoxetine and placebo, and fluoxetine versus placebo in peri- and postmenopausal women with moderate to severe depression scored by the 17-item HRSD.

Secondary aims are: (1) to assess the efficacy of IHT versus fluoxetine and placebo, and fluoxetine versus placebo using the BDI and GS’s score; and (2) to determine the safety of IHT versus fluoxetine and placebo, and fluoxetine versus placebo in peri- and postmenopausal women with moderate to severe depression.

## Methods/design

### Study design

The study will be a randomized, placebo-controlled, double-blind, double-dummy, three-arm parallel, superiority trial with a six-week study duration.

### Study setting

The study will be conducted in a public, academic and research hospital in Mexico City, the Juárez de México Hospital (JMH), which belongs to the Ministry of Health (MoH), the central authority in charge of health policies and design programs. The MoH provides health care to people without social security. JMH is an academic specialized hospital. Homeopathy is part of the National Health System regularized by the MoH. The outpatient service of homeopathy was established in JMH in 2004 and provides health care for climacteric stage women daily.

### Participant recruitment

The recruitment methods will include advertisements through the internet, local media and community groups, and liaisons with general practitioners, gynecologists, psychologists and allied health professionals. Posters with information about the study protocol will be posted at the study site; brochures will be distributed among the hospital population. Participants are currently being recruited starting in March 2012. The study is planned to end in December 2013.

### Eligibility criteria

The entry criteria for the study will be: (1) 40 to 65 years of age; (2) diagnosis of major depression according to the Diagnostic and Statistical Manual of Mental Disorders, 4th edition (DSM-IV); (3) moderate to severe depression according to the 17-item HRSD (14 to 24 score); (4) no current use of homeopathic treatment for depression or antidepressants or anxiolytic drugs for three months prior to study entry; (5) not taking psychotherapy for at least three months before screening; (6) no use of estrogens or other medications known to affect ovarian function for at least three months before screening; (7) early transition to menopause, defined by a change in cycle length of seven days or longer in either direction from the participant’s own baseline for at least two cycles; or late transition to menopause, defined as three to eleven months of amenorrhea; (8) postmenopausal stage defined by 12 months or more of amenorrhea; and (9) capability and willingness to give informed consent and to comply with the study procedures.

Exclusion criteria include: (1) pregnancy or breastfeeding; (2) other psychiatric disorders different from moderate to severe depression (severe depression, schizophrenia, psychotic disorders, bipolar affective disorders, suicide attempt, and so on); (3) alcohol or other substance abuse; (4) known allergy to fluoxetine; and (5) cancer or hepatic diseases.

### Types of interventions

**Figure 1:**
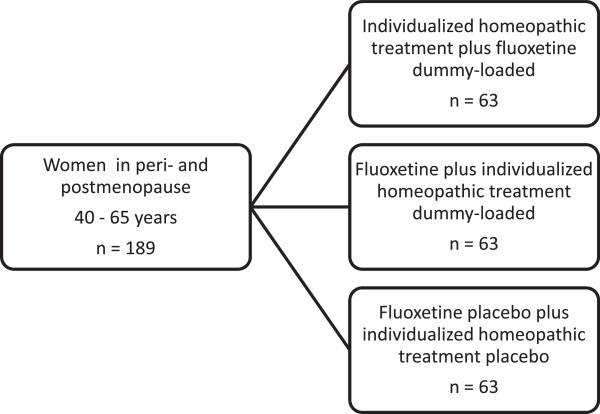
Flow chart of the study groups.

After inclusion, patients will be randomly assigned to either one of three groups illustrated in Figure 1: (1) IHT plus fluoxetine dummy-loaded; (2) fluoxetine (20 mg/day) plus IHT dummy-loaded; and (3) fluoxetine placebo plus IHT placebo.

The selection of the individualized remedy will be carried out after the case history by a certified medical doctor, specialized in homeopathy with 18 years’ experience in classical homeopathy. The case history will be based on Hahnemann’s methodology described in paragraphs 83 to 104 of Organon of Medicine, 6th edition. A complete medical history with clinical examination will also be done. All patients will have a full homeopathic case-taking including the collection of all the facts pertaining to the patient which may help in determining the totality of the symptomatology: past and present physical and emotional symptoms, family environment since childhood, stressful life events, marital satisfaction. The symptoms will be organized by hierarchy: mental, general and physical. In the first place, the strategy to choose the individualized remedy will be based on the most characteristic and clear mental symptoms. Secondly, general symptoms will be taken into account. A computerized version of the Synthesis Homeopathic Repertory 9.1 (Radar version 10) will be used to facilitate the prescription. Only one remedy will be prescribed at a time but it could be changed at every follow-up according to the patient’s symptoms.

C-potencies will be provided by Laboratorio Similia (Mexico City) and are manufactured according to the Mexican Homeopathic Pharmacopoeia and Hahnemann’s methodology. Each individualized homeopathic remedy will be prescribed in C-potencies (Appendix 1). Higher initial potencies will be tried, ranging from 30 to 200 C. The factors that influence the selection of the potency will include: clarity of mental symptoms, patient’s vitality and sensitivity, nature and kingdom (source) of medicine, chronicity and presence of any pathological disorder. A single dose of the individualized homeopathic remedy selected will be dissolved in a 60 ml bottle of 30% alcohol-distilled water. Patients will receive 15 drops PO two times per day following agitation for 10 days, plus fluoxetine-dummy loaded prescribed PO daily. A double-dummy technique with matching placebos for each active treatment will be applied, thus both placebos will seem identical to their corresponding *verum* formulations. Follow-up will be at weeks four and six after the first clinical interview.

Patients in the fluoxetine group will receive 20 mg/day PO plus IHT-dummy loaded. IHT-dummy loaded will be repeated at week four. Capsules of a generic fluoxetine will be provided by Laboratorio Similia (Mexico City). Placebo capsules will contain sucrose micro globules. Homeopathic placebo bottles will be filled with the same amount of 30% alcohol-distilled water. Patients will receive 15 drops PO of this solution two times per day following agitation for 10 days. The homeopathic placebo will be repeated at week four.

The third group will receive both fluoxetine and IHT placebos, as previously described.

### Criteria for discontinuing or modifying allocated interventions

Some adverse effects have been observed during fluoxetine treatment: lack of interest in sex, sexual dysfunction, nausea, insomnia, somnolence, anorexia, anxiety, asthenia, tremor, allergic skin reactions. If they are serious and/or result in interruption of treatment, they will be reported as adverse events.

During the IHT participants could have one of these reactions: (1) temporary intensification of symptoms before the condition improves, which is named ‘homeopathic aggravation.’ If it occurs at all it is usually mild and simply an indication that the body is resolving the problem in a natural and healthy way. If aggravation is too distressing it will be lessened by using frequent doses of the same remedy in a lower potency; (2) improving symptoms without aggravation; and (3) the appearance of new symptoms different from those on which the prescription was based. They will be reported as adverse events. They are rarely serious, but in that case, the IHT will be stopped. If the participant experiences the same symptoms of the remedy, the corresponding antidote will be prescribed.

Each participant will receive a report form on which to write daily any adverse event observed during the trial duration. Study participants will be retained in the trial whenever possible to enable follow-up data collection and prevent missing data.

### Adherence to interventions

To enhance the validity of the data, participants will return the unused capsules and bottles at each follow-up visit. Unused capsules will be counted and recorded on the appropriate case report form. Participants will be asked about any problems they are having taking their study treatment.

### Concomitant interventions

Some medications are prohibited during the study duration: triptans, tramadol, anxiolytic drugs, other serotonergic agents or antidepressants, as well as hormone replacement therapy. Psychotherapy is also forbidden during the study duration. Medication for diabetes and hypertension is allowed. The rescue intervention in case of a lack of efficacy in the IHT and placebo groups will be fluoxetine 20 mg/day; in the fluoxetine group, the rescue medication will be sertraline 25 mg/day.

### Participant retention

Plans to promote participant retention and complete follow-up will include: scheduling appointments and contacting patients by telephone.

### Outcomes

The primary efficacy outcome is the change from baseline in mean total depression score using the 17-item version of the HRSD at weeks four and six. The severity of symptoms will be assessed by a blinded investigator (clinical psychologist) from the JMH. The secondary outcomes are: change from baseline in mean total depression score using BDI (a self-reported scale) at weeks four and six; change from baseline in mean total score of GS at weeks four and six; responder rates (response rate: decrease of 50% or more from baseline score; remission rate: HRSD ≤7). The number and severity of all adverse events and homeopathic aggravations during the study period and 15 days after the final dose will be collected to determine the safety of fluoxetine and homeopathic medicines. An adverse event will be defined as any untoward medical occurrence in a subject without regard to the possibility of a causal relationship. Adverse events will be collected after participants have given consent and enrolled in the study and 15 days after study completion.

### Randomization

Participants are simple randomized in a 1:1:1 ratio using a computer-generated random allocation sequence, by a statistician not further involved in the study. Participants will be assigned in sequential order to the treatment groups. The randomization list will be kept strictly confidential.

### Allocation

#### Concealment mechanism and implementation

The principal investigator will enroll participants. Following inclusion, all patients will go through a full homeopathic case-taking and will receive a prescription of the individualized homeopathic medicine. Only one-third of the participants will actually receive a prescription. The research pharmacist will randomly deliver the treatment according to the allocation sequence in one of the three groups previously described. The randomization list will be sent to the research pharmacist at the start of the study. In case of emergency interventions, clinical worsening or adverse events, the pharmacist will inform the homeopathic doctor if the individual patient is taking homeopathy, fluoxetine or placebo, without disclosing the code.

### Blinding

Participants, the homeopathic doctor, the psychologist and the statistician will remain blinded to the identity of the three treatment groups until the end of the study. The psychologist will assess the severity of the symptoms and will keep the HRSD scores strictly confidential in a closed envelope at every follow-up until the end of the study.

### Sample size calculation

Sample size calculation was estimated using G*Power (available at the University of Düsseldorf: http://www.psycho.uni-duesseldorf.de/abteilungen/aap/gpower3/). The sample size calculation is based on a previous study protocol for a randomized controlled trial of homeopathy for depression published by Adler *et al.*[21]. We assumed that *verum* treatment is better than placebo by 2.7 ± 6.0 (mean ± standard deviation) HRSD score points after six weeks [21], corresponding to an effect size = 0.45 (largest difference between any two groups to be detected/expected within group standard deviation = diff/de). To detect an effect size = 0.45, in a three-group design (1:1:1), using F-Test, with a 5% risk of type 1 error (α) and 83% power, 63 patients per group are required considering also a 10% drop-out rate.

### Data collection

Study data will be collected at baseline, at every follow-up and 15 days after the completion of the study. Data will be collected from different sources: medical records, questionnaires (HRSD, BDI, GS) and report forms where participants will write daily any adverse event.

### Data management

All data will be entered electronically on data sheets designed for the study. The original study forms will be entered and kept on file at the JMH. Participant files are to be stored in numerical order in a secure and accessible place and manner. Participant files will be maintained in storage for a period of five years after completion of the study. All forms related to the study data will be kept in locked cabinets. Access to the study data will be restricted. A complete back-up of the data will be performed every month.

### Statistical analysis

All patients under randomization will be included in the primary efficacy population (intention-to-treat population), regardless whether or not they adhered to the treatment protocol or provided complete data sets. Only patients who withdraw their consent to use their personal data will be excluded from the analysis. The flow of participants through the trial will be presented in a Consolidate Standards of Reporting Trials (CONSORT) diagram.

First, the three groups will be compared in order to verify that there are no significant differences among them at baseline to confirm they are comparable after randomization. Demographic characteristics will be summarized using means and standard deviation for continuous data (that is, age) and relative frequencies for qualitative data (that is, marital status, comorbidities). The baseline demographic characteristics among groups will be compared with the use of the chi square test or by one-way independent measures of analysis of variance (ANOVA) as required.

We will compare: (1) IHT versus placebo; (2) fluoxetine versus placebo; and (3) IHT versus fluoxetine. The main statistical analysis will compare primary and secondary outcome measurements among groups at weeks four and six. The primary outcome (change in mean HRSD score) and secondary outcomes (change in mean BDI and GS scores) among groups at baseline and weeks four and six, will be analyzed by one-way ANOVA to provide a statistical test of whether or not the means of the three groups are all equal. The statistically significant one-way ANOVA result (*P* <0.05) suggests rejecting the global null hypothesis H_0_ (that the means are the same across IHT, fluoxetine and placebo groups). The Bonferroni *post-hoc* test will be used to determine which means differ among the groups. Responder rates will be compared among the groups using the chi square test. Statistical significance will be set at *P* <0.05 level for all analysis. Missing data will be handled by sensitivity analysis.

All patients who received at least one dose of the study drugs will be considered in the safety analysis. Adverse events will be translated to Medical Dictionary for Regulatory Activities terms (MeDRa terms), quantified and compared among the groups using the chi square test. Homeopathic aggravation will be compared among the groups also using the chi square test.

### Auditing

The Research and Ethics Committee of JMH, which is not involved in the study, will review the trial process every three months.

### Confidentiality

All study-related information will be stored securely at the study site. Participants’ study information will not be released outside of the study without the written permission of the participant.

### Regulatory and ethical approval

The trial protocol has been reviewed and approved by the Research and Ethics Committee of the Juárez de México Hospital (Submission Nº HJM 2030/12-A). This study is in compliance with the Helsinki Declaration and with the International Conference on Harmonisation (ICH) – Good Clinical Practice. Prior to undertaking any study related procedures, each participant will receive a verbal and written explanation of study aims, methods, potential hazards, and benefits from investigators and will provide written informed consent.

### Post-trial care

In the case of adverse events and after the completion of the study, participants may continue receiving health care at JMH.

## Discussion

For the first time this study evaluates both the specific effect of IHT using C-potencies versus fluoxetine and placebo in peri- and postmenopausal women with moderate to severe depression. Besides comparing homeopathic medicines versus placebo, the fluoxetine-arm will give more useful information about the effect of IHT versus an efficacious treatment for depression in a randomized controlled trial. It is an attempt to deal with the obstacles of homeopathic research due to the need of individual prescriptions in one of the most common psychiatric disorders. Homeopathy is frequently prescribed for climacteric symptoms including depression as it has been proven in some observational studies, but there is a lack of high-quality trials to prove its efficacy. This study protocol is based on: (1) CONSORT guidelines for reporting randomized trials with parallel groups; (2) the reporting data on homeopathic treatments (RedHot) supplement to CONSORT [32,33]; and (3) the Standard Protocol Items: Recommendations for Interventional Trials (SPIRIT) 2013 guidance for protocols of clinical trials [34].

It has been reported that the homeopathic consultation is in itself a therapeutic intervention working independently of the prescribed remedy [35], so all study participants will go through the same full homeopathic case-taking regardless of group allocation. Because placebo interventions are associated with mean response or remission rates of 35% [36,37], this trial includes a placebo-arm, so placebo effect can be ruled out.

Gibbons *et al.* reported that patients in all age and antidepressant groups have significantly greater improvement relative to placebo controls; and fluoxetine has been proven to be efficacious for depression in adults after six weeks of treatment [38], so it is expected that the fluoxetine-arm will result in significant differences versus placebo in this study. However, Kirsch *et al.* reported that, at present, it is becoming more difficult to prove that antidepressants actually work better than placebo in moderate to severe depression. It also has to be taken into consideration that the antidepressant-placebo difference seems to be smaller in the trials with moderately depressed participants [13]. Therefore, a fluoxetine-arm will allow us to detect if in Mexican women in the climacteric stage with moderate to severe depression, the difference in HRSD score between fluoxetine and placebo is as small as it has been shown in some meta-analysis [13].

Otherwise, different scenarios can result if no significant differences between IHT and placebo groups are found. In classical homeopathy, recovery requires the prescription of the individualized remedy at the adequate potency and dosage, so this is an important factor to be taken into consideration during the trial process because results may be biased. A negative result could be due to a mistaken election of the remedy and not because the IHT is inefficacious. Follow-up duration is also important. The selection of a suitable, individualized homeopathic medicine will not always be accomplished during six weeks of treatment, especially under double-blind conditions [21]. It has not been proved that six weeks of IHT is enough time to find a clinically relevant response in climacteric women with depression. In routine homeopathic consultation, a patient may require more than six weeks to recover from depression. However, for ethical reasons a longer follow-up is not possible because of the placebo group. Furthermore, homeopathic prescriptions may need to be modified depending on a patient’s individual response after homeopathic treatment is initiated. Once again, this could be problematic under double-blind conditions.

For ethical reasons women with severe depression will be excluded, so data of this trial will be able to prove an effective treatment in Mexican climacteric women with moderate to severe depression. Depression severity will be scored by the 17-item HRSD, which is a standardized instrument that has been used in many other clinical trials for depression. Another self-administered instrument, the BDI will also be used, so the results of the study might provide useful information in clarifying the controversy over the efficacy of homeopathy in depression. Use of these standardized instruments enables us to compare results with other trials published worldwide.

## Trial status

Participant recruitment began in March 2012.

## Appendix 1

C-potencies will be stored at Juárez de México Hospital. Medicines not listed can optionally be ordered and prescribed, as needed.

Actea racemosa, Aurum metallicum, Calcarea carbonica, Conium maculatum, Coffea cruda, Gelsemium sempervirens, Glonoinum, Helonias dioica, Kali carbonicum, Kali phosphoricum, Lachesis trigonocephalus, Lilium tigrinum, Lycopodium clavatum, Murex purpurea, Natrum muriaticum, Nux vomica, Phosphoricum acidum, Phosphorus, Platina, Pulsatilla nigricans, Sanguinaria canadensis, Sepia officinalis, Spigelia, Staphysagria.

## Abbreviations

ANOVA: analysis of variance; BDI: Beck Depression Inventory; CONSORT: Consolidated Standards of Reporting Trials; C-potencies: centesimal potencies; D-potencies: decimal potencies; DSM-IV: Diagnostic and Statistical Manual of Mental Disorders, fourth edition; FSH: follicle stimulating hormone; GS: Greene Climacteric Scale; HRSD: Hamilton Rating Scale for Depression; ICH: International Conference on Harmonisation; IHT: individualized homeopathic treatment; JMH: Juárez de México Hospital; MDD: major depressive disorder; MeDRa terms: Medical Dictionary for Regulatory Activities Terms; NICE: National Institute for Clinical Excellence; Q or LM potencies: quinquagintamillesimal; STRAW: Stages of Reproductive Aging Workshop.

## Competing interests

The authors declare that they have no competing interests.

## Authors’ contributions

ECMC, JAB and LAF participated in the design of the study. ECMC and JAB reviewed and discussed data for moderate depression, fluoxetine and homeopathy. ECMC, JAB and LAF made important contributions to the design of the trial, were substantially involved in drafting and revising the manuscript, and gave final approval of the submitted version of the document. All authors read and approved the final manuscript.

## Authors’ information

ECMC is a Homeopathic MD, MSc of Escuela Superior de Medicina, Instituto Politécnico Nacional. She was responsible for initiating the outpatient service of homeopathy in Juárez de México Hospital. JAB and LAF are full time professors and investigators of Escuela Superior de Medicina, Instituto Politécnico Nacional.
